# Anatomic factors influencing dimensions of soft tissue graft from the hard palate. A clinical study

**DOI:** 10.1002/cre2.298

**Published:** 2020-04-23

**Authors:** Khalid N. Said, Areej S. Abu Khalid, Fathima F. Farook

**Affiliations:** ^1^ College of Dentistry King Saud Bin Abdul Aziz University for Health Sciences Riyadh Saudi Arabia; ^2^ Department of Dental Services Ministry of National Guard‐Health Affairs Riyadh Saudi Arabia; ^3^ King Abdullah International Medical Research Center Riyadh Saudi Arabia; ^4^ Department of Dental and Oral Health Prince Sultan Military College of Health Sciences Dhahran Saudi Arabia

**Keywords:** masticatory mucosa, palatal thickness, transgingival probing

## Abstract

**Objectives:**

The aim of the present study was to measure the thickness of the palatal mucosa in a Jordanian (Middle Eastern) population as well as identify possible factors that may influence the thickness of palatal mucosa.

**Material and Methods:**

Sixty period on tally healthy subjects (29 males and 31 females) were selected. Fifteen measurement points were defined on the palate. The mucosal thickness in the hard palate was determined by “bone sounding” with a Hu‐Friedy® round periodontal probe.

**Results:**

The overall mean thickness of the palatal masticatory mucosa was 3.23 ± 0.47 mm.The mean thickness increased from the gingival margin to a more apical position irrespective of the tooth measured or side of the mouth in the following sequence: canine, second molar, first premolar, second premolar and lastly, the first molar. No significant difference between gender, smoking status, gingival phenotype andsides of the mouth with the thickness of palatal masticatory mucosa was determined. A significant difference between palatal shape and palatal gingival thickness was found.

**Conclusions:**

The most appropriate site for graft harvesting is the canine‐premolar area 8‐13 mm from the mid‐palatal aspect of each respective tooth in a Jordanian population. Except for the palatal shape, the side of the mouth, smoking, gender or gingival phenotype does not affect the graft harvest.

**Clinical Relevance:**

**Scientific Rationale for Study:**

Knowledge on the thickness of the masticatory mucosa is crucialin making decisions for surgical treatment modality and may affect surgical outcome. We measured the thickness of the palatal mucosa in a Jordanian population and identified possible influencing factors.

**Principal Findings:**

The thickness varied according to the teeth and the canine to premolar region was found to be the appropriate donor site.

**Practical Implications:**

This information on safe zone for graft harvest can guide the periodontist to make appropriate incisions and choose the appropriate location to obtain a graft of adequate thickness and dimensions.

## 1 INTRODUCTION

Periodontal plastic surgical techniques have evolved to meet the esthetic and functional demands of contemporary patients (Koke, Sander, Heinecke, & Muller, 2003; Yilmaz, Boke, & Ayali, 2015). Several surgical techniques have been proposed to enhance the gingival soft tissue volume and appearance (Yilmaz et al., 2015). The most common donor site for soft tissue grafts in the oral cavity is the hard palate and tuberosity (Ioannou et al., 2015). Attached keratinized mucosa, palatal to the maxillary premolars, is used to harvest full‐epithelialized free grafts or subepithelial connective tissue grafts for plastic surgery for oral and periodontal soft tissue augmentation procedures (Raetzke, 1985; Seibert, 1983; Sullivan, 1968; Zucchelli et al., 2020).

The thickness of the soft tissue graft plays an important role in the survivability of the graft, the healing modality and the clinical outcome of the mucogingival surgery (Kim & Neiva, 2015). The thickness of the tissue to be grafted from the donor site is an important factor in determining the appropriate treatment method and to predict the prognosis (Cairo, 2017; Stipetic, Hrala, & Celebic, 2005). Knowing the thickness of the palate would guide the periodontist to make appropriate incisions and, choose the appropriate location to obtain a graft of adequate thickness and dimensions. Several factors are believed to affect the thickness of the palatal gingiva including age, gender, race, smoking status, dentition, orthodontic treatment, systemic diseases, drugs, immunosuppression, and individual variations (Heil et al., 2018; Khatri et al., 2017; Manjunath, Rana, & Sarkar, 2015; Stipetic et al., 2005).

Conflicting findings regarding the palatal mucosal thickness has been reported in literature (Barriviera, Duarte, Januário, Faber, & Bezerra, 2009; Gupta, Jan, Behal, Mir, & Shafi, 2015; Heil et al., 2018; Müller, Heinecke, Schaller, & Eger, 2000; Song et al., 2008; Wara‐aswapati, Pitiphat, Chandrapho, Rattanayatikul, & Karimbux, 2001).

To our knowledge, none of the previous studies evaluated patients from Arab or Mediterranean ethnicities. The lack of information on regional differences in the thickness of the palatal masticatory mucosa prompted us to carry out this study. The objective of the study was to measure the thickness of the palatal mucosa in a Jordanian (Middle Eastern) population as well as identify possible factors that may influence the thickness of palatal mucosa.

## 2 MATERIALS AND METHODS

The study design was a prospective study. A consecutive sampling method was used to recruit 60 systemically healthy Jordanian participants who attended the Periodontics and Oral and Maxillofacial Surgery clinic at the Dental Teaching Center, Jordan University of Science and Technology. Participants who had periodontally healthy full maxillary dentition (except for third molars) and scheduled for anesthetized procedures of the hard palate, were included. The study protocol was approved by the Institutional Review Board of the University.

Participants with a history of any disease or surgery in the palate or tuberosity, presence of any dental appliances in the upper arch, previous orthodontic treatment, extracted or congenitally missing premolars, using medication that would affect the periodontal soft tissue, malposition, or malalignment of the maxillary posterior teeth and those who declined participation were excluded. Informed consent was obtained after explaining the study protocol. The researcher completed a questionnaire collecting information regarding smoking status, palate anatomy, type of occlusion as well as dental and medical history.

**FIGURE 1:**
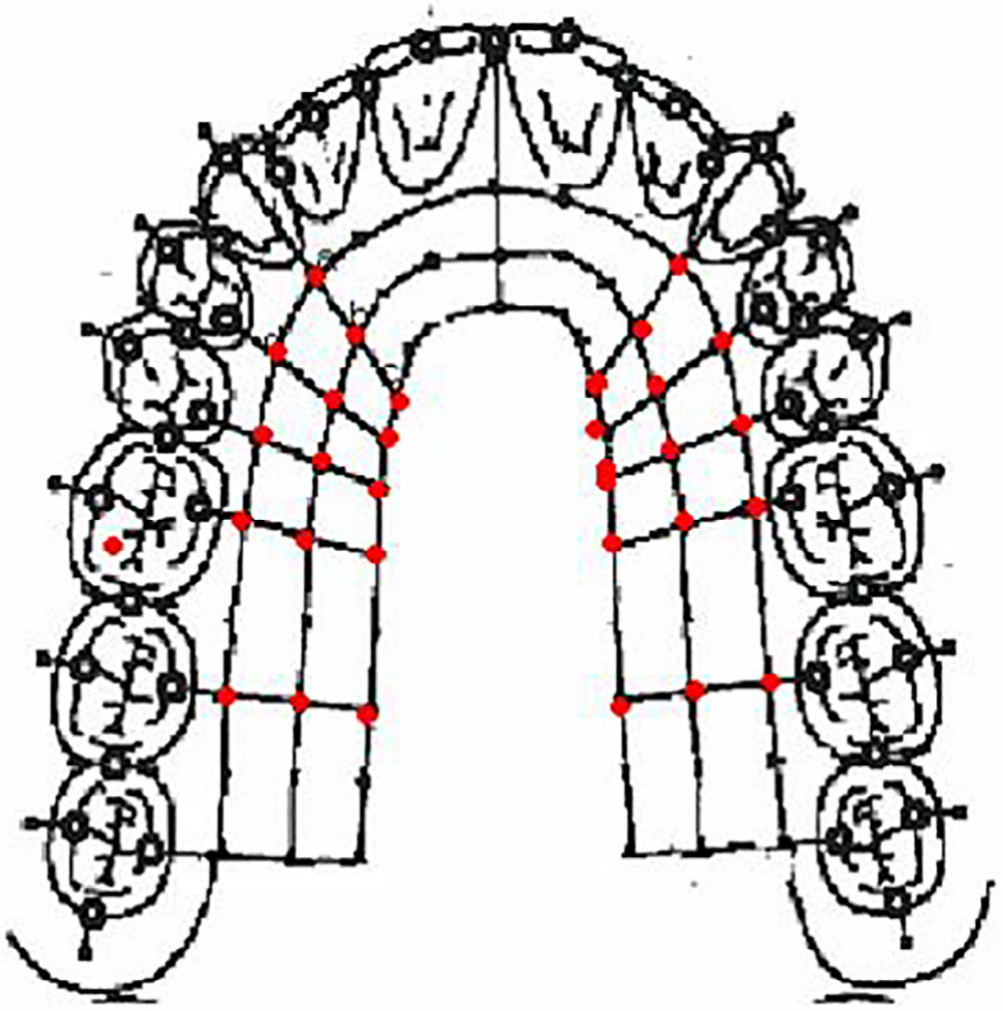
Measurement points: Five vertical lines perpendicular to the gingival margin between the canine and second maxillary molar were defined to determine 15 standard measurement points (marked in red) in the hard palate. Three horizontal lines running parallel to the gingival margin were established starting at the mid‐palatal aspect of the canine and ending over the palatal root of the second molar. The distances from the gingival margin to the horizontal lines were 3 mm (Point a), 8 mm (Point b), and 13 mm (Point c), respectively

Five vertical lines perpendicular to the gingival margin between the canine and second maxillary molar were defined to determine 15 standard measurement points for the hard palate (Figure 1). Three horizontal lines running parallel to the gingival margin were established starting at the mid‐palatal aspect of the canine and ending over the palatal root of the second molar. The distance from the gingival margin to the horizontal lines were 3 mm (point a), 8 mm (point b), and 13 mm (point c), respectively. Five points were defined on each of the horizontal lines intersecting with the vertical lines, constituting five positions, each located at the level of a tooth position. The first position was determined at the mid‐palatal aspect of the canine, second at the mid‐palatal aspect of the first premolar, third at the mid‐palatal aspect of the second premolar, fourth over the palatal root of the first molar, fifth over the palatal root of the second molar. Measurements were done bilaterally.

**FIGURE 2:**
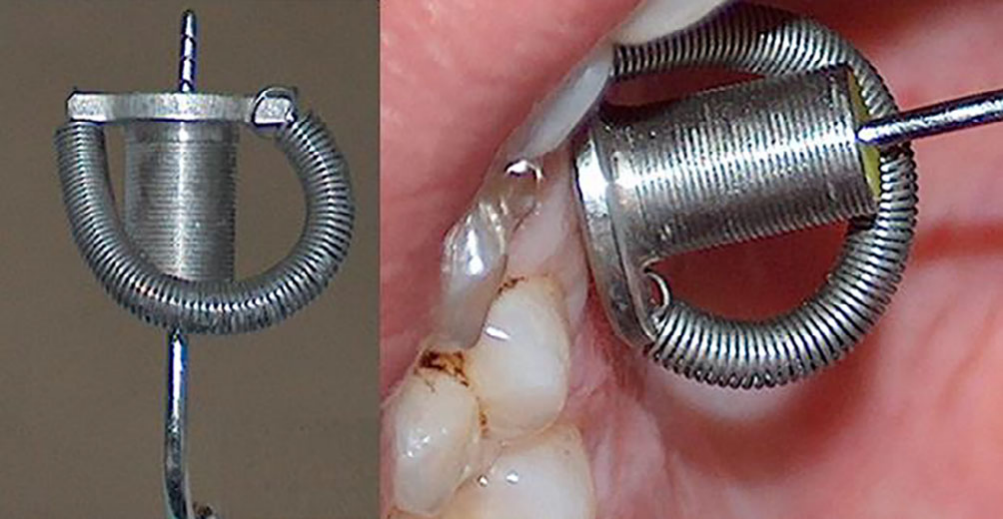
(a) Probe with stopper: view of the Hu‐Friedy® periodontal probe with the stopper in place. Note that the stopper does not slip even if held upside down. This assures that the measurement taken stays accurate when removed from the patient's mouth. (b) Sounding technique: the probe inserted into the palatal gingival mucosa 3 mm from the gingival margin with the stopper adjusted into position. Note that the long axis of the probe is perpendicular to the palate and the horizontal wing of the stopper is in direct contact with the mucosa

The hard palate was anesthetized with a spray, followed by an Octacaine injection with an epinephrine vasoconstrictor (2%) of 1:100,000. A block injection was administered in the region of the greater palatine foramen with 0.1 ml of anesthetic and in the region of the incisive foramen with 0.05 ml. The anesthetic was injected slowly over 30 s and measurements were taken 10 min later. The mucosal thickness in the hard palate and tuberosity was determined by “bone sounding” with a Hu‐Friedy® round periodontal probe (round with 1 mm increments). Each probe was equipped with a special stopper (Impla System drill stopper®) originally manufactured for an implant 2.0 mm twist drill stabilization (Figure 2a). The stopper was modified to stabilize the periodontal probe and to ensure that the probe inserted vertically in the palatal mucosa. The stopper was filled with hard acrylic resin and two rubber stoppers were inserted at the top and the end of the cylinder.

In the transverse dimension, the periodontal probe was positioned with the tine at a right angle to the corresponding gingival margin of each tooth to be measured at the center of the tooth. This assured the correct alignment to lines a–c. In the sagittal dimension, an imaginary line was drawn through the tips of the canine cusp and the lingual cusps of the first and second premolars to create a parallel line with the measurement points at 3, 8, and 13 mm from the second premolar. An imaginary line through the mesiolingual cusps of the first and second molars defined the parallel line for the first and second molars. This was done to establish reproducible palatal points in the transverse and sagittal dimensions.

After securing the stopper to the probe, the probe tip was inserted into the mucosa and forced down to the bone at each measurement site (Figure 2b). The probe was held perpendicular to the palate and the angulation was confirmed by keeping the stopper wing parallel to and as close as possible to (most of the time in direct contact) the surface of the mucosa. The probe was then removed from the mucosa and the reading recorded at the corresponding site on the diagram.

If any rugae were present in the probing area, the “valley” point instead of the “hill” point was defined as the measurement site. At such points, the stopper was moved away to enable recording of the thickness. The readings on the probe were approximated to the lowest reading when the tissue thickness did not match the probe marks. When the measurement was not exactly on the millimeter line of the probe, the value was rounded up to the next half millimeter.

Apart from the above outcome measurement, the records of the following explanatory variables smoking status, gingival phenotype (thick and thin phenotypes), palatal shape (high and shallow palate), and gender were also recorded. For the gingival phenotype, the thickness of the tissue in the bucco‐palatal dimension was taken by inserting a probe into the mid‐buccal sulcus of the respective tooth. If the probe was visible through the tissue it was classified as thin, otherwise thick. The palatal shape was reproduced for recording from alginate impression casted in dental stone. For this, an impression of each subject was made with irreversible hydrocolloid impression material to obtain study casts poured with dental plaster.

All measurements were performed twice by the same investigator (intra‐rater reliability ICC ≥0.995; *p*‐value <.001). The average of the two measurements was taken as the final measurement for the thickness at each site.

Analysis was performed using statistical software (SPSS®, version 23.0). Mean, median, with *SD*s of masticatory mucosa thickness in different measurement points were presented using descriptive statistics. The relation between palatal gingival thickness with other variables gender, side, gingival phenotype, smoking, and palatal shape were analyzed using independent *t* tests. Pearson correlation analysis was performed to see the relation between mean palatal thickness at the left and right sides. Statistical hypotheses tests were two‐tailed comparisons and the criteria for statistical significance were accepted at the probability level *p* < .05.

## 3 RESULT

Gender was equally distributed in the sample (*n* = 60) with 90% nonsmokers.

### 3.1 Palatal gingival thickness per site and tooth

**FIGURE 3:**
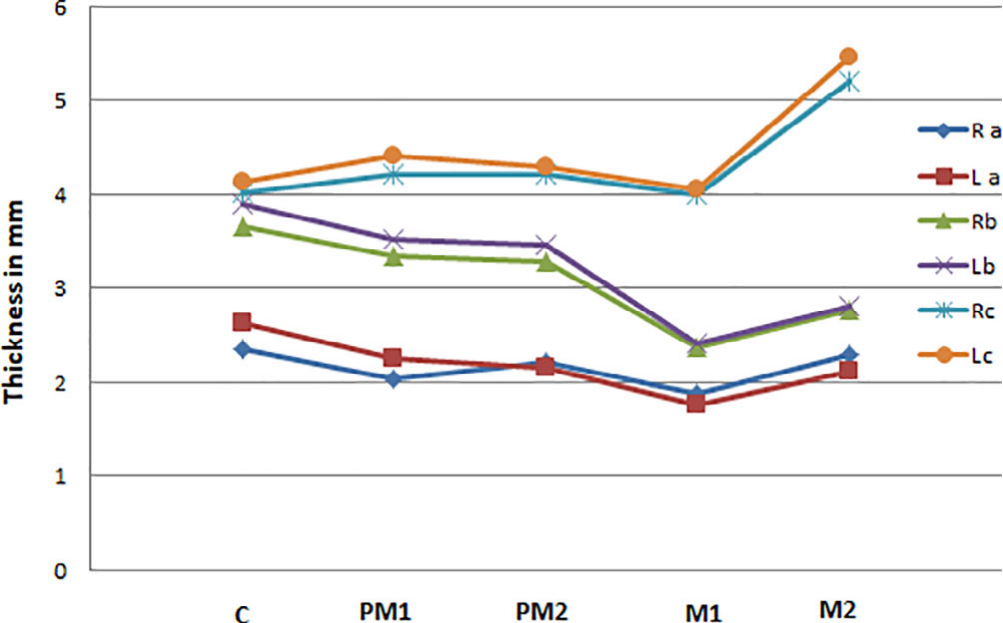
Relationship of the thickness of the mucosa (mm) at the measurement Points a, b, and c of right and left sides of the canine (C), first premolar (PM1), second premolar (PM2), first molar (M1), and second molar (M2)

The mean thickness of the masticatory mucosa at each measurement point is presented in Table 1. The mean thickness increased from point a through to Point c irrespective of the tooth measured or side of the mouth. The thickest areas were 13 mm from the mid‐palatal aspect of the following teeth in a decreasing order: upper second molar, upper first premolar, upper second premolar, upper canine, and finally the upper first molar (Table 1). At 3 mm from the mid‐palatal aspect of the corresponding teeth, gingival thickness was highest at the canine site and lowest at the first molar area (Table 1). A similar trend was found at 8 mm from the gingival margin but with a much sharper reduction at the first molar area and an increase in the first molar area close to the level of the thickness in the second premolar area (Table 1). At 13 mm, the thickness increased from the canine posteriorly, almost plateaued at the premolar area, and reduced only slightly at the first molar area. The highest reading was measured at the second molar area, which was significantly different to all the other readings (Table 1 and Figure 3). The mean thickness of the palatal mucosa descends in the following sequence: canine, second molar, first premolar, second premolar and lastly, the first molar.

**TABLE 1:** Tooth‐wise comparison of thickness of palatal mucosa at Points a, b, and c

Tooth	a	b	c	Range (min–max)	Average
Canine	2.51 ± 0.56	3.75 ± 0.65	4.07 ± 0.56	2.0–5.0	3.44 ± 0.52	
First premolar	2.15 ± 0.47	3.43 ± 0.70	4.29 ± 0.76	2.0–5.3	3.29 ± 052	
Second premolar	2.17 ± 0.49	3.36 ± 0.85	4.25 ± 0.73	2.0–5.2	3.26 ± 0.54	
First molar	1.82 ± 0.61	2.38 ± 0.73	4.01 ± 1.24	1.3–5.5	2.74 ± 0.63	
Second molar	2.22 ± 0.91	2.74 ± 1.03	5.29 ± 1.29	1.2–6.5	3.41 ± 0.82	
Range (min–max)	1.1–4.4	1.6–5.3	2.0–7.1	–	–	
Average	2.18 ± 0.41	3.15 ± 0.59	4.38 ± 0.67	–	3.23 ± 0.47	

*Note:* The measurement points reflect the distances from the gingival margin to the horizontal lines: Point a at 3 mm, Point b at 8 mm, and Point c at 13 mm from the gingival margin.

### 3.2 Palatal gingival thickness on the right and left sides

The graph in Figure 3 reflects the close association between the data curvatures from both sides of the palate, showing a pattern of symmetry of bilateral readings at each point (a, b, and c). There was a significant positive correlation for the mean palatal thickness between the right and left Point a, b, and c pairs except for the first premolars (*p* = .11) and the second premolars (*p* = .06) at Point a. No significant difference was found between the right and left side readings except between the right and left canines at Points a (*p* = −.01) and b (*p* = .04).

Evaluating the correlation of the palatal gingival thickness of the right and left sides *per tooth* using a *t*‐test, a significant correlation was found for all teeth (*p* < .01) and no significant difference (*p* = .12) was found except for the canine (*p* < .01) and the first premolar (*p* = .03). Overall, the mean thicknesses of palatal gingiva in the right side (3.22 ± 0.51 mm) and in the left side (3.29 ± 0.50 mm).

### 3.3 Palatal gingival thickness and smoking/gender

No significant association was found between gender, smoking status with gingival thickness regardless of the teeth and the measurement points (Table 2).

**TABLE 2:** Mean thickness of palatal mucosa with *SD*s with respect to gender, smoking status, gingival phenotype, and palatal shape

Variable	Categories	Mean ± *SD* (mm)	*p*‐Value
Gender	Male	3.22 ± 0.43	.85
	Female	3.24 ± 0.52	
Smoking status	Smoker	3.14 ± 0.47	.61
	Nonsmoker	3.24 ± 0.48	
Gingival phenotype	Thick	3.30 ± 0.50	.07
	Thin	3.06 ± 0.50	
Palatal shape	High‐narrow	3.33 ± 0.46	.03
	Wide‐shallow	3.06 ± 0.46	

### 3.4 Palatal gingival thickness and gingival phenotype

With regard to gingival phenotype, 66.7% of the sample consisted of thick, less scalloped gingival phenotypes with 33.3% consisting of thin, more scalloped gingival phenotypes.

No significant difference was found between gingival phenotype and palatal gingival thickness (*p* = .07). However, at each measurement site, a significant difference was found between gingival phenotype and palatal thickness in the following points at 13 mm from the gingival margin: upper right canine (*p* = .04), upper right first premolar (*p* < .01), upper right second premolar (*p* = .02), upper left canine (*p* = .01), and the upper left first premolar (*p* = .002).

### 3.5 Palatal gingival thickness and palatal shape

With regard to the maxillary palatal shape, 63.3% of the sample exhibited a narrow, high palate, with 36.7% exhibiting a wide, shallow palate. A significant difference between palatal shape and palatal gingival thickness was found (*p* = .03; Table 2).

## 4 DISCUSSION

The majority of the sample (91.4%) was between 13 and 30 years old, which is the most frequent age group for mucogingival surgeries (Moura et al., 2017; Zucchelli & De Sanctis, 2000). Half of the sample was female, all were systemically healthy and 90% were nonsmokers (Table 1). Our exclusion criteria ensured minimal effects of potentially confounding factors of the palatal thickness. Based on literature, the thickness of masticatory mucosa has been evaluated by invasive and noninvasive methods, such as using injection needles or probes (Claffey & Shanley, 1986; Goaslind, Robertson, Mahan, Morrison, & Olson, 1977; Olssoin, Lindhe, & Marinello, 1993; Pendleton, 1934), histologic sections (Anderegg, Metzler, & Nicoll, 1995), cephalometric radiographs (Östlund, 1958), and ultrasonic devices (Baldi et al., 1999; Daly, 1971; Jan, 1987; Lytle, 1957; Terakura, 1986; Uchida, Kobayashi, & Nagao, 1989). The ultrasonographic method of assessing gingival thickness has several disadvantages (Daly, 1971), including the relative unavailability of the instrument, difficulty in maintaining the directionality of the transducer, and (Eger, Müller, & Heinecke, 1996) non‐reliable results when the thickness of gingiva exceeds 2–2.5 mm (Vandana & Savitha, 2005).

To overcome these disadvantages, “bone sounding” was used in the current study. However, measurements of palatal thickness using bone sounding could be inaccurate due to the added thickness caused by the anesthetic solution. Necessary precautions were taken to minimize this unintended volume increase by using a minimal amount of anesthetic solution, slow injection, waiting for a minimum of 10 min after injections before probing and by giving block injections in the incisive papilla and greater palatine foramina (Müller, Schaller, & Eger, 1999). We used a stopper that was designed to assure the correct positioning of the probe perpendicular to the evaluation point at the palate and exact probe readings after removal from the tissue since the stopper does not slide on the probe due to the cylinder fill (acrylic fill and rubber stoppers).

The assessment of potential donor sites in the palate revealed that the thickest mucosa was 13 mm from the gingival margin of the upper second molar. However, due to the close proximity of the greater palatine neurovascular bundle, this site is not a suitable donor site since most of the complications are associated with the underestimation of anatomic structures, such as the greater palatine artery (GPA; Tavelli, Barootchi, Ravidà, Oh, & Wang, 2019). The thinnest mucosa in the hard palate is in the region of the upper first molar, which can be explained by the position and curvature of the palatal root of the maxillary first molar excluding the site as a possible donor site. It is not advisable to harvest soft tissue grafts beyond this structure to avoid increasing the risk of accidental damage to the GPA or its branches.

Our study revealed that the thickest mucosa is within 8–13 mm from the gingival margin in the region of the upper second molar followed by the canine and then the premolar. The findings of this study is on par with previous literature, reporting that the palatal side of the maxillary first molar has the thinnest overlying mucosa and recommending the canine‐premolar area for graft harvesting because the mucosa is the thickest in the hard palate region (Müller, Schaller, Eger, & Heinecke, 2000; Stipetic et al., 2005; Studer, Allen, Rees, & Kouba, 1997; Wara‐aswapati et al., 2001).

Several harvesting approaches have been proposed for soft tissue grafts such as trap‐door, epithelialized gingival graft, split thickness, de‐epithelialized techniques, single incision, parallel incision, and many others. And different thicknesses have been suggested for the various grafting procedures. A few of the optimal thickness of palatal mucosa required for various grafting procedures demonstrated by different studies are: 0.9 mm (James & McFall Jr, 1978), 2.0 mm (Miller, 1982), 1.5–2.0 mm (Goldman, Isenberg, & Shuman, 1976; Goldman et al., 1976; James & McFall Jr, 1978; Miller, 1982), 1.0–1.5 mm(Maynard, 1977), and 0.8–1.3 mm (Soehren, Allen, Cutright, & Seibert, 1973). A free palatal graft of 0.9 mm thickness proved to be functionally sufficient regardless of whether they healed on denuded alveolar bone or a periosteal bed (James & McFall Jr, 1978). The ideal thickness of the graft should be 1–1.5 mm thick. However, regardless of the palatal harvesting approach, it can be recommended that 8–13 mm from the gingival margin at the canine‐premolar area is the most suitable area for the graft harvest.

Our mean thickness was 3.23 ± 0.46 mm, which is higher than findings from previous studies (Müller, Heinecke, et al., 2000; Studer et al., 1997; Wara‐aswapati et al., 2001). The difference could be attributed to differences in race, thickness of the sub mucosa or the method of measurement. Palatal gingival thickness can be underestimated due to unavoidable compression of the mucosa with the ultrasound probe tip (Schulze, Ćurić, & d'Hoedt, 2002) and the dentures in edentulous patients (Uchida et al., 1989). Shculze et al. compared the measurements obtained by an ultrasonic device with needle probing (Schulze et al., 2002). Our findings are similar to those obtained by the latter method only in the molar region. The measurements reported by Uchida et al. (1989) using an ultrasonic device are similar to the current study at the 13 mm points in the molar and premolar areas.

There was no significant relation between gender and palatal gingival thickness in our study. However, females showed a slightly higher mean thickness than the males. Our results are in agreement with two studies that used a bone sounding technique (Studer et al., 1997; Wara‐aswapati et al., 2001). However, Wara‐aswapati et al. reported a slightly lower mean thickness for the females compared to the males, which was not significant. The authors used a comparable sample size and the same number of measurement points but at slightly different distances from the gingival margin. Our study is in contrast to several previous studies reporting a thinner mucosa for females compared to males. The difference with the Ostlund study might be explained by the differences in sites chosen for measurement, measurement techniques or measurement error as the study was conducted with edentulous patients who may have been wearing dentures, which could have an influence on gingival thickness (Östlund, 1958). Menopause and aging atrophy are important factors causing ridge reduction (Lammie, 1956). Overall, the differences between this study and others could be attributed to the lack of unification in the selection criteria, method of measurement, type of measuring device, sample size, and selected measurement sites and different significance levels (Müller et al., 1999; Müller, Heinecke, et al., 2000; Östlund, 1958; Seibert, 1983; Stipetic et al., 2005; Vandana & Savitha, 2005).

No significant difference (*p* = .61) was found between smoking status and gingival thickness. Smokers represented only 10.3% of our subjects. Our findings are consistent with those of Müller, Schaller, et al. (2000) who reported the same results using clinical parameters. A higher epithelial thickness histologically was observed in smokers independent of the gingival health (Villar & de Lima, 2003). This may be due to an actual lack of a relationship or due to the small sample size of smokers (10.3%) in our study.

Our study showed no significant difference (*p* = .12) between the right and left sides of the mouth which was also reported by Uchida et al. (1989). The symmetry facilitates surgical decision making, irrespective of the sides.

No significant difference was found between gingival phenotype and palatal gingival thickness (*p* = .07). This is not in accordance with the results found by Müller, Schaller, et al. (2000), but may be due to a different classification of gingival phenotype and different measurement points as they included the mandible. A unified classification for gingival phenotype is not yet available to enable comparable analysis.

A significant difference between palatal shape and palatal gingival thickness was found (*p* = .03). Jaws with high‐narrow palates showed a significantly higher (*p* = .03) mean palatal gingival thickness (3.33 ± 0.46 mm) compared to jaws with wide, shallow palates (3.06 ± 0.46 mm). However, this method of visual assessment is subjective.

Recommendations have been suggested for specific palatal locations to harvest donor graft tissue from the site closest to the tooth to any area extending between the distal of the second molar and the canine or even up to the lateral incisor. These recommendations were based on uniformity of the thickness, vessels emergence, width and thickness of the gingiva, and the amount of sub mucosa (Brasher, Rees, & Boyce, 1975; Soehren et al., 1973). It should also be noted that type of soft tissue graft (either FGG or subepithelial connective tissue graft) may have a different effect on the vascular injury of the palate (Tavelli et al., 2020).

According to Brasher et al. (1975), grafts that are too thin fail to produce an adequately increased zone of keratinized gingival and if too thick, it will result in an exaggerated tissue profile in the area. An average thickness of 1 mm was suggested as an acceptable thickness (Brasher et al., 1975). For thin grafts when 1 mm is an adequate donor tissue thickness, the entire area from the canine to the second premolar is a potential donor site. However, when thicker connective tissue grafts are required, any region between the canine and second premolar could be used as donor graft tissue, if it is more than 8 mm from the gingival margin. The measurements at the 3 mm site for the canine‐premolar region consist of connective tissue and epithelium, although above 2 mm.

Our finding suggests that the area of the canine and premolars 8–13 mm from the gingival margin is the best site for harvesting palatal soft tissue grafts with less risk of endangering the greater palatine bundle but with an absolute risk of an aesthetic unacceptable outcome particularly in free gingival grafts due to presence of the rugae. However, clinicians should be cautious when involving the canine since the branches of the GPA tend to become more coronal due to variations in the topography of the GPA (Yu et al., 2014). Although the thickest area is adjacent to the upper second molar, the presence of glandular tissue at this site and the fact that 13 mm from this tooth is close to the soft palate posteriorly as well as the greater palatine foramen, which could endanger the vessels arising from there. This, together with the position and curvature of the palatal root of the upper first molar, limits the possible graft donation to the area beyond 9 mm.

However, due to the close proximity of the greater palatine neurovascular bundle, this site is not a suitable donor site. The thinnest mucosa in the hard palate is in the region of the upper first molar, which can be explained by the position and curvature of the palatal root of the maxillary first molar excluding the site as a possible donor site. It is not advisable to harvest soft tissue grafts beyond this structure to avoid increasing the risk of accidental damage to the GPA or its branches.

## 5 CONCLUSION

Within the limitations of this study, it can be concluded that the possible most appropriate site for graft harvesting is the canine‐premolar area 8–13 mm from the mid‐palatal aspect of each respective tooth in a Jordanian population. However, beyond 13 mm from the gingival margin at the distal aspect of the second premolar, there is an increased risk due to the presence of greater palatine neurovascular bundle. The side of the mouth, smoking, gender, or gingival phenotype does not affect the graft harvest. Further studies are required to explore the potential risks of harvesting soft tissue grafts from areas deeper in the palate.

## CONFLICT OF INTEREST

The authors declare no conflict of interest, financial or otherwise.
